# Long‐term impact of a behavioral weight management program on depression and anxiety symptoms: 5‐year follow‐up of the WRAP trial

**DOI:** 10.1002/oby.23570

**Published:** 2022-10-27

**Authors:** Rebecca A. Jones, Julia Mueller, Stephen J. Sharp, Simon J. Griffin, Amy L. Ahern

**Affiliations:** ^1^ MRC Epidemiology Unit University of Cambridge Cambridge UK; ^2^ Primary Care Unit, Department of Public Health and Primary Care University of Cambridge Cambridge UK

## Abstract

**Objective:**

Behavioral weight management programs may support short‐term mental health; however, limited evidence reports the long‐term impacts. This study investigated the impact of behavioral weight management programs on depression and anxiety symptoms at 5 years from baseline.

**Methods:**

The Weight loss Referrals for Adults in Primary care (WRAP) trial randomized 1267 adults with BMI ≥ 28 kg/m^2^ to a brief intervention (BI) or commercial behavioral weight management program (WW; formerly Weight Watchers) for 12 or 52 weeks (CP12 and CP52, respectively). Linear regression was used to separately compare 5‐year changes in depression and anxiety symptoms (by Hospital Anxiety and Depression Scale) between randomized groups, adjusting for baseline depression/anxiety symptoms, gender, and research center.

**Results:**

A total of 643 (51%) participants attended the 5‐year study follow‐up visit. There was no evidence of a difference between the randomized groups for 5‐year changes in depression (BI: −0.08 ± 3.29; CP12: 0.02 ± 3.01; CP52: −0.09 ± 3.41) or anxiety (BI: 0.16 ± 3.50; CP12: −0.05 ± 3.55; CP52: −0.66 ± 3.59) symptoms.

**Conclusions:**

This study found no evidence that commercial weight management programs differed in 5‐year changes in depression and anxiety symptoms, compared with BI. These are average effects; some individuals experienced increases or decreases in symptoms. Future research should investigate who is at most risk of mental health declines and investigate how to support them. Future trials should transparently report long‐term mental health outcomes to strengthen understanding.

**Study Importance**

**What is already known?**

- Although evidence suggests that behavioral weight management programs may support mental health in the short term, there is a lack of trials measuring and reporting mental health in the longer term.
- It is important to understand how programs impact mental health in the long term to ensure appropriate care and treatment are provided to participants.

**What does this study add?**

- We found no evidence to suggest that, on average, attending a commercial weight management program had greater benefits for depression and anxiety symptoms compared with a brief intervention.
- Importantly, we also found no evidence that, on average, attending a commercial weight management program had worse impacts on long‐term depression or anxiety symptoms than a brief intervention.

**How might these results change the direction of research or the focus of clinical practice?**

- The study findings may provide reassurance to both participants and providers that engaging with a behavioral weight management program is unlikely to have negative long‐term impacts on mental health.
- We encourage future research to measure and transparently report the long‐term effects of weight management programs on mental health to strengthen the evidence base.

## INTRODUCTION

Overweight and obesity impact more than 60% of the adult population in England [1], with prevalence increasing worldwide. The health consequences of living with obesity include increased risk of type 2 diabetes, stroke, some cancers (e.g., endometrial, esophageal, kidney cancer), and cardiovascular disease [2, 3, 4, 5, 6, 7], as well as mood disorders, binge eating disorder, and psychological distress [8, 9]. Behavioral weight management programs are the most common form of obesity treatment, and there is good evidence that they can achieve improvements in physical health [2, 3, 4, 5, 6, 7, 10, 11]. A recent systematic review found that these programs are also supportive of some aspects of mental health at the end of the program compared with inactive/minimal interventions or “usual care.” Specifically, the review found improvements in depression symptoms, mental health‐related quality of life, and self‐efficacy [12] and found no evidence of a detrimental impact on any aspect of mental health. However, it also identified a lack of research assessing the long‐term impacts of these programs on mental health.

The end of a behavioral weight management program is a pivotal point in a participant's weight management journey. Adults with obesity rarely receive formal support after completing a behavioral weight management program, and the absence of support can exacerbate feelings of stress, anxiety, and psychological distress. In addition, research reports a high rate of weight regain upon completion of a behavioral weight management program [13], potentially causing feelings of failure, self‐blame, and shame. Furthermore, there are some concerns that participating in a weight management program can have long‐lasting detrimental effects on mental health, with some research highlighting a worsening in mental health for a proportion of participants after weight loss efforts [14, 15]. It is hypothesized that this may be related to increased focus on dietary restrictions and body shape/image and increased awareness of the risks of living with obesity. It is important to understand how programs impact mental health in the long term to ensure appropriate care and treatment are provided to participants. This may be in the form of better preparing participants for the program to end, providing longer‐term support, referring to external sources of long‐term care, or adaptations to provider training.

Weight management trials seldom measure mental health measures at time points greater than 12 months from baseline [12], potentially because of lack of funding for long‐term follow‐up, high rates of study withdrawal, and concerns about participant burden. Of the limited available evidence, Rubin and colleagues found no evidence of a difference in health‐related quality of life at 2 years from attending a 24‐month behavioral weight loss intervention [16], and similarly, Alfaris and colleagues found no difference in mood at 2 years from attending a 24‐month practice‐based behavioral weight loss intervention [17]. Although these trials provide measures at 2 years from baseline, they also provide interventions with a 2‐year duration, which means that the follow‐up measures do not represent sustained effects in the long term after the end of the intervention. This limits our understanding of the long‐term impacts on mental health and inhibits the further development of weight management programs to effectively support the mental health of adults with obesity.

The Weight loss Referrals for Adults in Primary care (WRAP) trial, however, is uniquely positioned to investigate the impact of these programs on long‐term mental health [18]. In 2014, the WRAP trial recruited and randomized adults with overweight or obesity to a commercial behavioral weight management program (WW; formerly Weight Watchers) for 12 weeks or 52 weeks or to a brief intervention (BI; a 32‐page booklet containing weight management strategies). Participants completed follow‐up assessments, including mental health measures, at 3 months, 1 year, 2 years, and 5 years from baseline [18]. Although at 3 months from baseline, previous WRAP analyses found small reductions in depression symptoms in those attending WW compared with BI, there was no evidence of greater improvements for symptoms of depression and anxiety and, notably, also no evidence of a detrimental impact of the commercial program on symptoms of depression and anxiety at 1 year and 2 years from baseline [19]. The current study examines the long‐term impact of these programs on depression and anxiety symptoms at 5 years from baseline.

To our knowledge, this paper is the first to investigate the impact of a commercial behavioral weight management program on symptoms of depression and anxiety at 5 years from baseline using data from a randomized controlled trial. We aimed to evaluate the impact of referral to a 52‐week or 12‐week weight management program on symptoms of depression and anxiety at 5 years compared with BI. We also aimed to compare the impact of the 52‐week program and 12‐week program.

## METHODS

### Study design

This is a secondary data analysis of the WRAP trial, a randomized controlled trial comparing three arms: (1) BI, (2) 12‐week commercial weight loss program (CP12), and (3) 52‐week commercial weight loss program (CP52). Ethical approval for up to 2‐year post‐randomization assessment was received by National Research Ethics Service (NRES) East of England Cambridge East and local approvals from NRES Committee North West Liverpool Central and NRES Committee South Central Oxford. Ethical approval for 5‐year post‐randomization assessment was received from West Midlands Coventry and Warwickshire Research Ethics Committee. The original trial (ISRCTN82857232) and 5‐year follow‐up (ISRCTN64986150) were prospectively registered with ISRCTN. Participants who met eligibility criteria and gave informed consent were randomly assigned to an intervention arm on a 2:5:5 ratio. More detailed trial methods are reported elsewhere [18].

### Participants

Eligible participants (adults with body mass index [BMI] of 28 kg/m^2^ or greater, residing in the United Kingdom) were identified and recruited by local primary care practices across England. Individuals were excluded from study participation if they met one or more of the following: planned or current pregnancy in the next 2 years, previous or planned bariatric surgery, current weight loss program, non‐English‐speaking or communication needs that would preclude them from understanding the study materials and interventions, and general practitioner‐defined inappropriateness for participation (e.g., patients who are violent/terminally ill/have a history of an eating disorder). Further details of inclusion and exclusion criteria can be found elsewhere [18].

### Intervention

Participants randomly assigned to CP12 or CP52 were provided with vouchers to attend a weekly local WW meeting for the duration of the intervention they were assigned to (i.e., 12 or 52 weeks). Participants were provided with a unique code to access digital tools for the duration of their assigned intervention.

### Control group

Participants assigned to the BI control group received a 32‐page printed British Heart Foundation booklet containing self‐help weight management strategies [20]. Research staff read a scripted booklet introduction to the participant.

### Outcomes

Study participants completed assessments at baseline and 3, 12, 24, and 60 months. The study outcomes were change from baseline to 5 years in depression and anxiety symptoms, measured by the Hospital Anxiety and Depression Scale (HADS) [21]. The HADS measure is a validated screening tool with 14 items (7 anxiety; 7 depression) scored on a Likert scale from 0 to 3 [21, 22, 23]. The scale produces symptom scores for anxiety and depression that range from 0 to 21, with a score equal to or greater than 11 representing moderate to severe symptoms of depression and/or anxiety [21, 22, 23]. The scale cannot provide a clinical diagnosis of anxiety or depression [21, 22, 23]. Symptoms of anxiety and depression are recommended to be measured by HADS in the recent core outcome set for behavioral adult weight management interventions [24].

A minimal important difference (MID) for change in the HADS score has not been determined in a population of adults with overweight and obesity. However, in alternative populations (cancer, cardiovascular disease, bronchiectasis, and chronic obstructive pulmonary disease), it has most commonly been defined as a change of 2 points [25, 26, 27, 28, 29]. Therefore, in this analysis we defined MID to be a change of ±2 points in HADS score for depression or anxiety symptoms.

### Statistical analysis

Stata software (version 16; StataCorp LLC) was used for statistical analysis. Descriptive summary statistics were calculated for baseline characteristics by randomized group, with numbers of participants, means, and standard deviations (SD) presented for continuous variables and numbers and proportions of participants presented for categorical variables.

The primary analysis used linear regression to separately compare 5‐year change in depression and anxiety symptoms between the three randomized groups, adjusting for baseline depression and anxiety symptoms, as well as the randomization stratifiers (gender and research center), i.e., ANCOVA. The null hypothesis of no difference between the three groups was tested using an *F* test. Estimates and 95% confidence intervals (CI) of differences comparing 52‐week program versus BI, 52‐week program versus 12‐week program, and 12‐week program versus BI group were obtained from the model. As no significant difference was found between the randomized groups, in accordance with the prespecified statistical analysis plan, a secondary analysis was conducted to compare the 52‐week program and the other groups (12‐week program and BI) combined. In all analyses, participants were included in the group to which they were randomized, based on the intention‐to‐treat principle.

## RESULTS

### Participant characteristics

Participants characteristics at baseline are presented in Table 1. Study participants had a mean BMI between 34.43 (± 4.63) and 34.68 (± 5.39), with a similar proportion of participants self‐reporting taking antidepressant medication at baseline across study arms (19%–23%). At baseline, study participants had similar levels of depression (mean [SD], BI: 5.58 [3.77]; CP12: 5.24 [3.38]; CP52 5.20 [3.64]) and anxiety (BI: 7.25 [4.29]; CP12: 6.89 [3.97]; CP52 7.29 [4.09]) symptoms across groups. The number of participants attending study follow‐up visits at 60 months was 643 (51%) (BI: 95 [45%]; CP12: 285 [54%]; CP52: 263 [50%]).

**TABLE 1:** Participant characteristics at baseline, presented by randomized group

		BI (*n* = 211)	12‐week CWMP (*n* = 528)	52‐week CWMP (*n* = 528)
Age (y), mean ± SD		51.91 ± 14.07	53.63 ± 13.26	53.30 ± 13.96
Female		143 (68%)	357 (68%)	359 (68%)
BMI, mean ± SD		34.43 ± 4.63	34.68 ± 5.39	34.45 ± 5.05
Self‐reports taking antidepressant medication		26 (19%)	81 (21%)	81 (23%)
Anxiety symptoms, mean ± SD^a^		7.25 ± 4.29	6.89 ± 3.97	7.29 ± 4.09
Depression symptoms, mean ± SD^a^		5.58 ± 3.77	5.24 ± 3.38	5.20 ± 3.64
Ethnicity	White or White British	181 (86%)	480 (91%)	475 (90%)
	Asian or Asian British	9 (4%)	11 (2%)	15 (3%)
	Black or Black British	5 (2%)	12 (2%)	6 (1%)
	Mixed or multiple ethnic group	4 (2%)	4 (1%)	7 (1%)
	Other, missing, or prefer not to say	12 (6%)	21 (4%)	25 (4%)
Index of Multiple Deprivation decile	1 (least deprived)	16 (8%)	45 (8.52%)	44 (8.37%)
	2	8 (4%)	16 (3%)	26 (5%)
	3	12 (6%)	38 (7%)	32 (6%)
	4	17 (8%)	37 (7%)	38 (8%)
	5	31 (15%)	49 (9%)	50 (10%)
	6	19 (9%)	61 (12%)	57 (11%)
	7	32 (15%)	67 (13%)	63 (12%)
	8	19 (9%)	69 (13%)	75 (14%)
	9	12 (6%)	51 (10%)	47 (9%)
	10 (most deprived)	45 (213%)	95 (18%)	94 (18%)
Level of education	None	7 (3.57%)	25 (5.27%)	27 (5.78%)
	GCSE or equivalent	55 (28%)	153 (32%)	155 (33%)
	A‐Level or equivalent	53 (27%)	95 (20%)	110 (24%)
	Postsecondary study	10 (5%)	14 (3%)	10 (2%)
	University degree or equivalent	48 (24%)	108 (23%)	97 (21%)
	Higher degree or equivalent	23 (12%)	79 (17%)	68 (15%)
Household income	£0–£19,999	65 (33%)	125 (25%)	138 (28%)
	£20,000–£49,999	66 (33%)	173 (34%)	176 (35%)
	£50,000+	41 (21%)	91 (18%)	84 (17%)
	Do not know/prefer not to say	27 (14%)	113 (23%)	101 (20%)

*Note*: Data are number and percentage, unless otherwise stated.

Abbreviations: BI, brief intervention; CWMP, commercial weight management program; GCSE, General Certificate of Secondary Education.

^a^ Depression and anxiety symptoms are measured by the Hospital Anxiety and Depression Scale (HADS) measure, which is a validated screening tool with 14 items (7 anxiety; 7 depression) scored on a Likert scale from 0 to 3. The scale produces symptom scores for anxiety and depression that range from 0 to 21, with higher scores indicating higher depression/anxiety symptoms and with a score equal to or greater than 11 representing moderate to severe symptoms of depression and/or anxiety.

### Impact on depression and anxiety symptoms at 60 months

#### Impact on depression symptoms at 60 months

Mean changes in depression symptom scores at 60 months were −0.08 (SD 3.29) after BI, 0.02 (SD 3.01) in the 12‐week program, and −0.09 (SD 3.41) in the 52‐week program (Table 2). On average, changes in depression symptoms at 60 months were less than the MID of 2 and, therefore, did not represent meaningful change. Histograms (in online Supporting Information) highlighting the distribution of changes in depression symptoms from baseline to 3, 12, 24, and 60 months showe that, at all time points, some people in all groups experienced an improvement or decline in their mental health. A proportion of participants in each group experienced a meaningful change in mental health; 22 BI participants (33%), 62 CP12 participants (30%), and 56 CP52 participants (28%) experienced a meaningful decrease in symptoms of depression at 60 months, whereas 11 BI participants (17%), 37 CP12 participants (18%), and 42 CP52 participants (21%) experienced a meaningful increase in symptoms of depression at 60 months (Table 3).

**TABLE 2:** Changes in depression and anxiety symptoms from baseline to 60 months

	Mean change in mental health from baseline to 60 months (± SD)			Estimated differences between groups (95% CI)			
	BI	CP12	CP52	CP12 vs. BI	CP52 vs. BI	CP52 vs. CP12	BI and CP12 vs. CP52
Depression symptoms	−0.08 (± 3.29) *n* = 66	0.02 (± 3.01) *n* = 206	−0.09 (± 3.41) *n* = 202	0.07 (−0.76 to 0.89)	−0.06 (−0.89 to 0.77)	−0.13 (−0.71 to 0.45)	−0.11 (−0.65 to 0.43)
Anxiety symptoms	0.16 (± 3.50) *n* = 66	−0.05 (± 3.55) *n* = 206	−0.66 (± 3.59) *n* = 206	0.07 (−0.98 to 0.85)	−0.59 (−1.51 to 0.33)	−0.52 (−1.16 to 0.12)	−0.54 (−1.14 to 0.06)

*Note*: Analyses were adjusted for baseline mental health, gender, and research center.

Abbreviations: BI, brief intervention; CP12, 12‐week commercial weight management program; CP52, 52‐week commercial weight management program.

**TABLE 3:** Proportion of participants by randomized group experiencing a decrease, no change, or increase in symptoms of depression or anxiety at 60 months from baseline

Change in symptoms of depression or anxiety symptoms at 60 months from baseline		Number of participants (%)		
		BI	CP12	CP52
Depression symptoms	Decrease (≤ −2 units)	22 (33%)	62 (30%)	56 (28%)
	No change (> −2 and <2 units)	33 (50%)	107 (52%)	104 (51%)
	Increase (≥ +2 units)	11 (17%)	37 (18%)	42 (21%)
Anxiety symptoms	Decrease (≤ −2 units)	19 (29%)	63 (31%)	62 (30%)
	No change (> −2 and <2 units)	31 (47%)	102 (50%)	107 (52%)
	Increase (≥2 units)	16 (24%)	41 (20%)	37 (18%)

Abbreviations: BI, brief intervention; CP12, 12‐week commercial weight management program; CP52, 52‐week commercial weight management program.

We conducted ANCOVA to determine the difference between BI, CP12, and CP52 on the change in depression symptoms between baseline and 60 months, controlling for baseline depression and anxiety symptoms, gender, and research center. There was no evidence of a difference between randomized groups in change in depression symptoms (*F*(2) = 0.10, *p* = 0.91; *n* = 474).

Pairwise comparisons showed no difference between BI and CP12 (0.07; 95% CI: −0.76 to 0.89) or CP52 (−0.06; 95% CI: −0.89 to 0.77) or between CP12 and CP52 (−0.13; 95% CI: −0.71 to 0.45). Participants in the 52‐week behavioral program did not differ from the participants in the other groups (BI and 12‐week behavioral program) combined in changes to depression symptoms from baseline to 60 months (−0.11; 95% CI: −0.65 to 0.43) (Table 2).

#### Impact on anxiety symptoms at 60 months

Mean changes in anxiety symptom scores at 60 months were 0.16 (SD 3.50) after BI, −0.05 (SD 3.55) in the 12‐week program, and −0.66 (SD 3.59) in the 52‐week program (Table 2). On average, changes in anxiety symptoms at 60 months were less than the MID of 2 and, therefore, did not represent meaningful change. Histograms (in online Supporting Information) highlighting the distribution of changes in anxiety symptoms from baseline to 3, 12, 24, and 60 months showed that, at all time points, some people in all groups experienced an improvement or decline in their mental health. A proportion of participants in each group experienced a meaningful change in mental health; 19 BI participants (29%), 63 CP12 participants (31%), and 73 CP52 participants (36%) experienced a meaningful decrease in symptoms of anxiety at 60 months, whereas 16 BI participants (24%), 41 CP12 participants (20%), and 24 CP52 participants (12%) experienced a meaningful increase in symptoms of anxiety at 60 months (Table 3).

We conducted ANCOVA to determine the difference between BI, CP12, and CP52 on the change in anxiety symptoms between baseline and 60 months, controlling for baseline depression and anxiety symptoms, gender, and research center. There was no evidence of a difference between randomized groups in change in anxiety symptoms (*F*(2) = 1.55, *p* = 0.21; *n* = 474).

Pairwise comparisons showed no difference between BI and CP12 (−0.07; 95% CI: −0.98 to 0.85) or CP52 (−0.59; 95% CI −1.51 to 0.33) or between CP12 and CP522 (−0.52; 95% CI: −1.16 to 0.12). Participants in the 52‐week behavioral program did not significantly differ from the participants in the other groups (BI and 12‐week behavioral program) combined in changes to anxiety symptoms from baseline to 60 months (−0.54; 95% CI: −1.14 to 0.06) (Table 2).

## DISCUSSION

In this study, we aimed to assess the impact of commercial behavioral weight management programs on depression and anxiety symptoms at 5 years (60 months) from baseline. We found no evidence to suggest that, on average, attending a commercial weight management program has greater benefits for depression and anxiety symptoms compared with BI. Importantly, we also found no evidence that, on average, attending a commercial weight management program has worse impacts on long‐term depression or anxiety symptoms than BI.

Study findings should be interpreted with consideration to the large proportion of missing data. High proportions of missing data can reduce generalizability of the study findings, and potential reasons for the large proportion of missing data may include diminishment in interest and engagement in the trial in the longer term, and perceived inconvenience of completing follow‐up visits many years after completing the intervention. Despite this, the WRAP trial has a smaller attrition rate than is common in weight loss trials [30, 31, 32], and best efforts were made to transparently and clearly report missingness. In future trials, additional engagement strategies should be considered to improve participant retention at longer‐term study follow‐up visits.

A recent systematic review reported small benefits for some aspects of mental health at the end of a behavioral weight management program and highlighted the lack of evidence reporting the impacts on mental health in the longer term [12]. In previous WRAP analyses, Heath and colleagues found no evidence of harm or benefit to depression or anxiety symptoms at 1 and 2 years from baseline as a result of attending the commercial weight management programs [19]. Similarly, in the current analyses, we found that, on average, participants across all randomized groups did not experience meaningful changes (as defined by MID) in depression and anxiety symptoms from baseline to 60 months. However, these findings are average effects and may not represent the experiences of all program participants. We found that a proportion of participants in all randomized groups experienced a meaningful increase or decrease in depression and anxiety symptoms. Future research should look to identify those experiencing an increase in depression or anxiety symptoms and consider the support that may be most effective to minimize this risk. This may be achieved by identifying the program components most and least supportive of participant mental health to inform the design of future weight management programs.

The primary aim of a behavioral weight management program is to produce a meaningful reduction in body weight, with any changes in mental health considered secondary impacts or unintended benefits/consequences. As these programs are not designed to produce long‐term improvements in mental health, it is unsurprising we found no difference between randomized groups for change in depression and anxiety symptoms at 5 years from baseline. However, previous research has found that both adults with obesity and health care providers believe that weight management services do not sufficiently support mental health [33], suggesting that programs may benefit from adapting the development process to intentionally benefit mental health.

Strong evidence shows that weight gain/regain is common after program end, and this can result in a decline in mental health with participants reporting feelings of failure, blame, and shame [13]. We found that, on average, this behavioral weight management program did not result in long‐term increases in depression and anxiety symptoms, despite weight regain [34]. This may provide reassurance to both participants and providers that engaging with a behavioral weight management program is unlikely to have negative long‐term impacts on mental health.

To our knowledge, this paper is the first to investigate the impact of behavioral weight management programs on depression and anxiety symptoms at 5 years from baseline. The paper is strengthened by inclusion of minimal exclusion criteria, maximizing the generalizability of the study population at baseline. The trial had a reasonable rate of participant follow‐up, which is above average when compared with similar trials [30, 31, 32].

This analysis was limited by high levels of missing data, which meant it was not possible to perform subgroup analyses to assess if the impact of the interventions was different across particular groups, such as gender, education, or ethnicity. Despite prespecifying a sensitivity analysis using multiple imputation, we also prespecified that this would not be appropriate if the level of missingness were 25% or more, and would not be necessary if the level of missingness were less than 5%. A recently published secondary data analysis of the WRAP trial found that those reporting poorer mental health at baseline were less likely to attend the 5‐year follow‐up visits. Although the magnitude of the association was small, the findings of the current study should be interpreted with consideration that the samples may not represent the true range of participants' mental health experiences [35]. Future research should seek to implement strategies to improve data completion.

This analysis was further limited by the mental health measures included in the WRAP trial; the HADS only measures anxiety and depression symptoms, and a wider range of mental health measures such as distress, loneliness, disordered eating, and body image concerns would enable a broader understanding of the impact of these programs on mental health. It is important to understand the impact of weight management programs on a range of mental health outcomes as benefits in one domain may not be reflected in another; for example, the long‐term effects on anxiety and depression symptoms do not inform of the effects on stress and self‐esteem.

The generalizability of the study findings may be impacted by the participant eligibility criteria. Specifically, participants were ineligible for participation in the trial if they were deemed inappropriate for inclusion by their general practitioner. Among other reasons, this included a diagnosis of a severe mental illness. As such, the study findings may not reflect the true range of mental health experiences in those living with overweight or obesity. The generalisability of the study findings is further limited as the participant sample was largely made up of White, highly educated, females.

## CONCLUSION

On average, there was no evidence of a difference between the 12‐week weight management program, 52‐week weight management program, or brief intervention for changes in depression and anxiety symptoms at 5 years from baseline. Furthermore, there was no evidence that behavioral weight management programs cause harm to long‐term mental health. However, it is important to note that these are average effects and they do not rule out long‐term impacts of these programs in some people or on other aspects of mental health. Future research should aim to measure and transparently report the long‐term effects of weight management programs on a range of mental health outcomes and should seek to identify the participant and intervention characteristics that predict changes in mental health.

## AUTHOR CONTRIBUTIONS

RAJ designed the study, analyzed and interpreted the data, and drafted the manuscript. JM and SJS contributed to the design of the study and interpretation of the data and critically reviewed the manuscript. SJG provided critical appraisal of the study and manuscript. AA designed and supervised the study, interpreted the data, and critically appraised and reviewed the manuscript. All authors read and approved the final manuscript.

## FUNDING INFORMATION

The 5‐year follow‐up of the WRAP trial was funded by the National Institute for Health Research (NIHR) under its Programme Grants for Applied Research Programme (RP‐PG‐0216‐20010). The original WRAP trial was funded by the National Prevention Research Initiative through research grant MR/J000493. The intervention was provided by WW (formerly Weight Watchers) at no cost via a Medical Research Council (MRC) Industrial Collaboration Award. RAJ, ALA, SJG, and SJS are supported by the MRC (Grant MC_UU_00006/6). The University of Cambridge has received salary support in respect of SJG from the National Health Service in the East of England through the Clinical Academic Reserve.

## CONFLICT OF INTEREST

RAJ, JM, and SJS declared no conflict of interest. ALA and SJG are the chief investigators on two publicly funded (MRC, NIHR) trials in which the intervention is provided by WW at no cost.

## Supporting information


**Appendix S1** Supporting information

## Data Availability

The data set analyzed during the current study is not publicly available. Participant consent allows for data to be shared in future analyses with appropriate ethical approval, and the host institution has an access policy (https://www.mrc-epid.cam.ac.uk/wp-content/uploads/2019/02/Data-Access-Sharing-Policy-v1-0_FINAL.pdf) so that interested parties can obtain the data for replication or other research purposes that are ethically approved. Data access is available from the host institute (datasharing@mrc-epid.cam.ac.uk).
