# Supplementary material for: Long‐term impact of a behavioral weight management program on depression and anxiety symptoms: 5‐year follow‐up of the WRAP trial
Source: Obesity (Silver Spring). 2022 Oct 27;30(12):2396–403. doi: 10.1002/oby.23570 (PMC9828709; doi:10.1002/oby.23570)
Supplement: Supplementary file 1 — Appendix S1 Supporting information [file OBY-30-2396-s001.docx]

**Long-term impact of a behavioural weight management programme on depression and anxiety: 5-year follow up of the WRAP trial. APPENDIX 1.**

Rebecca A. Jones^1^ (rj397@cam.ac.uk), Julia Mueller^1^, Stephen J. Sharp^1^, Simon J. Griffin^1,2^, Amy L. Ahern^1^

^1^ MRC Epidemiology Unit, University of Cambridge, Cambridge, UK.

^2^ Primary Care Unit, Department of Public Health and Primary Care, University of Cambridge, Cambridge, UK.

*Figures 1-8. Histograms of change in anxiety and depression from baseline to 3-, 12-, 24-, and 60-months.*
